# Twenty Minutes with Emphysema

**DOI:** 10.1089/pmr.2020.0048

**Published:** 2020-06-04

**Authors:** Paul C. Rousseau

**Affiliations:** Charleston, South Carolina, USA.

The room is cold and dark, and smells of cigarettes. The only sound is the hiss of oxygen. I flip a light switch; his eyes squint. He's sitting on a couch wearing tattered pajamas with burn spots on the sleeves and legs. He stands to greet me. His legs buckle and he falls back.

I pull a kitchen chair next to him. He removes his nasal prongs, turns off the oxygen, and strikes a match. A blue haze wafts upward. Unfiltered Pall Malls. Government warnings, struggled breaths, and four dollar packs; nothing has stopped the aphrodisia of a cigarette. “I crave the damn things, but I know they're bad for me. It's like I'm addicted.”

“When I was young, life seemed unlimited. Time had no end.” He takes a quick puff. His face reddens. He coughs a deep, rumbling cough, veins bulging in his neck. He coughs and coughs. I stand and cradle his shoulders. He spits a thick glob of mucous into a vomit basin. I wonder whether the puff was worth it. He pulls an ashtray from an end table and snuffs the cigarette. “I only take one puff from each cigarette, it's all I can handle.”

He scrunches his legs together and rocks back and forth. “It's the coughing, always makes me have to pee.” I ask where his urinal is; he doesn't know. He stands and shuffles with a walker; I hold his arm and haul an oxygen tank behind. He rests his hands on the bathroom sink, looks in the mirror, and turns his head side to side, as if to be sure he's still alive. Rivulets of sweat trickle down his brow. “I get so hot breathing; that's why the house is so cold.” He takes his pajama top off. Bags of skin hang like curtains; bones rise like periscopes. Swaths of bruises color his arms. “I look pretty bad, don't I?” His lips twist into a grimace.

“I cough most days, sometimes all day; it's the phlegm.” He tells me it's usually white or grey, with an occasional splatter of blood. “Probably some cancer down there.” He sits on the commode and leans forward, his breathing a muddle of short puffs scattered with gulps and gasps. “Most days I feel empty,” he says, “like a gutted fish. No appetite, no nothing.” I nod, sadly.

He finishes and we return to the living room. He plops down on the couch, his body so slight he barely leaves an impression. He asks that I open the blinds. He glances out the window, his milk-flecked pupils roaming the street. “My days are nothing more than catching my next breath.” He grunts. “That's a helluva life, isn't it?” He removes the nasal prongs, turns off the oxygen, and lights another cigarette. I ask if he's ever forgotten to remove his oxygen before he smokes. “Never. I saw a guy forget once at the VA hospital. His whole head went up in flames. You don't forget something like that.” He takes a puff and pushes the cigarette into the ashtray. He coughs until he expels another glob of mucous. This one has a small blood clot. He leans back. His brow relaxes, his eyes close. He looks pensive. “It's getting close.” “What is?” I ask. “The end of my unlimited life.” My head slumps. We sit in a brief silence. Then he opens his eyes, removes the nasal prongs, turns off the oxygen, and lights another cigarette.

